# Isolated trigeminal nerve palsy with motor involvement as a presenting manifestation of multiple sclerosis in an equatorial region – a case report

**DOI:** 10.1186/1755-7682-5-17

**Published:** 2012-05-30

**Authors:** Eranda C Ratnayake, Manjula Caldera, Priyankara Perera, Ranjani Gamage

**Affiliations:** 1 Institute of Neurology, The National hospital of Sri Lanka, (Regent Street), Colombo, Sri Lanka; 2 Institute of Neurology, The National hospital of Sri Lanka, (Regent Street), Colombo, Sri Lanka; 3 Institute of Neurology, The National hospital of Sri Lanka, (Regent Street), Colombo, Sri Lanka

## Abstract

**Introduction:**

Isolated cranial nerve palsies are considered to be an uncommon presenting feature of multiple sclerosis. Involvement of the trigeminal nerve, particularly its motor component as part of a clinically isolated syndrome of multiple sclerosis has rarely been reported in equatorial regions and no cases have been described in Sri Lanka thus far.

**Case Presentation:**

We report a case of isolated right sided trigeminal nerve palsy (Motor and Sensory) in a 34 year old previously well lady from urban Sri Lanka who was found to have characteristic lesions on Magnetic Resonance Imaging highly suggestive of multiple sclerosis.

**Conclusions:**

Multiple sclerosis should be considered in the differential diagnosis of patients who present with isolated cranial nerve palsies. Clinicians should have a high index of suspicion when evaluating such patients especially in low prevalence regions close to the equator. Early recognition and treatment of such a “Clinically Isolated Syndrome” may prevent early relapse.

## Introduction

Despite being a relatively common disease in western countries, multiple sclerosis remains a rare entity in equatorial regions including Sri Lanka [1]. It is known to manifest in many forms including retrobulbar neuritis, cerebellar syndromes and transverse myelitis. An isolated cranial nerve palsy is considered to be a rare presenting sign of multiple sclerosis [2-4]. The pathogenesis of cranial nerve involvement in multiple sclerosis is not well described but it is commonly associated with brain stem demyelination [5]. The initial presenting manifestation of relapsing multiple sclerosis has been recently described as the “clinically isolated syndrome” (CIS) [6]. Thus, isolated cranial nerve palsies with characteristic imaging patterns would now fall in to this category.

## Case presentation

A 34 year old previously well lady from urban Sri Lanka presented to the Institute of Neurology at the National Hospital of Sri Lanka with a one month history of numbness of the right side of her face and difficulty chewing from the right side of her mouth. She described the onset of the symptoms as sudden as it was present on awakening from an uninterrupted sleep the previous night. The symptoms had persisted over a month without much progression. She had received treatment from a general practitioner but her symptoms had not improved. She did not have any diplopia or blurred vision, painful eye movements, hearing impairment, dysphagia or dysarthria. She did not complain of limb weakness or unsteadiness with tendency to fall. She did not complain of shooting pain on the right side of the face triggered by touch and has had no previous facial rashes. She denied sexual promiscuity. She had lived in Sri Lanka all her life and has not had any foreign travel.

**Figure 1:**
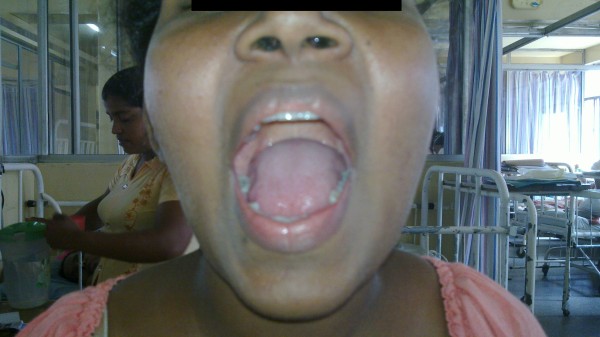
Deviation of the mouth to the right side on opening the jaw.

Neurological examination revealed an alert lady who was oriented to time, place and person. There was no facial asymmetry or drooling of saliva and no obvious facial rashes. On asking the patient to open her mouth there was slight jaw deviation to the right side (Figure 1). Palpation of the Temporalis and Masseters did not reveal any wasting. Sensory examination revealed impaired pain sensation on the right side of the face involving the Ophthalmic, Maxillary and Mandibular divisions of the trigeminal nerve conforming to the characteristic onion skin distribution. The corneal reflex on the right side was also absent. All other cranial nerve examination was normal including the adjacent nerves. Opthalmoscopic examination did no reveal optic atrophy or papillitis. There was no limb involvement with normal Corticospinal, Spinothalamic and Posterior column pathway examination including preserved reflexes and there were no demonstrable Cerebellar signs.

**Figure 2:**
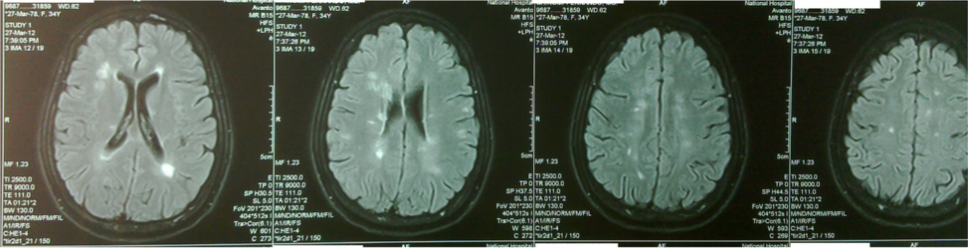
T2WI MRI brain images reveal multiple, hyperintense, periventricular deep white matter lesions highly suggestive of multiple sclerosis.

**Figure 3:**
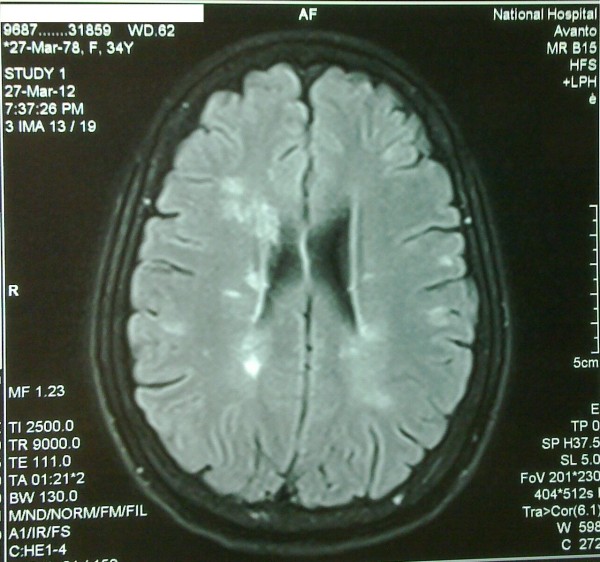
Close up image of T2WI MRI Brain image of multiple periventricular hyperintense lesions.

Magnetic Resonance Imaging (MRI) of the brain and spinal cord was subsequently performed and it revealed multiple, hyperintense (Both T2WI and T2 FLAIR), periventricular lesions in the deep white matter conforming to the characteristic Dawson’s Finger appearance which is highly suggestive of multiple sclerosis (Figures 2-3). There were also similar lesions in the right cerebellar peduncle. The spinal cord was free of lesions. Cerebrospinal fluid analysis revealed normal protein levels with no cells. The presence of oligoclonal bands could not be analyzed as this facility is unavailable at our institution and was unaffordable to the patient to be done in the private sector. Visual and Brainstem evoked potential studies were carried out and revealed normal results. All other basic biochemistry results were normal including normal inflammatory markers.

She was treated with intravenous Methylprednisalone 1 g daily for 3 days followed by oral Prednisalone 50 mg daily (1 mg/kg/day) with plans to gradually taper off the treatment later. She was advised on the possibility of further neurological deficits in the future and asked to adhere to the follow up programme.

## Discussion

Isolated cranial nerve palsies may be a presenting manifestation of multiple disease processes including cerebral vasculitis, basal meningitis and many other inflammatory conditions of the brainstem. Demyelinating diseases including multiple sclerosis may rarely present with isolated cranial nerve palsies as well [2-4]. When cranial nerves are affected in isolation the trigeminal nerve has been found to be the most frequently affected in some studies [7]. Due to its low prevalence in equatorial regions, multiple sclerosis is rarely considered in the differential diagnosis of isolated cranial nerve palsies. Furthermore due to the limited availability of MRI facilities in most parts of the region many patients with such presentations will be misdiagnosed or remain unrecognized.

A recent meeting of experts on multiple sclerosis coined the term “Clinically Isolated Syndrome” for the initial presenting manifestation of the disease [6]. It is reasonable to state that the patient described in this report falls in to this category. The importance of identifying patients with CIS is to prognosticate future relapses and the severity of the illness as well as identifying patient who require early disease modifying treatment. It has been shown that patients with CIS and a positive baseline MRI, such as in our patient have an 8 fold risk of developing a further attack over time. We treated our patient with Corticosteroids over Interferon β-1b due to the availability and cost effectiveness of the former treatment. Close follow up is required as the extensive lesions on MRI suggest an early relapse.

The pathogenesis of isolated cranial nerve palsies in multiple sclerosis remains inconclusive. Brainstem demyelination has been suggested to be the mechanism in some reports [5] but the MRI of our patient did not reveal any brainstem lesions. The possibility of a demyelinating process of the nerve itself should be considered in this case.

## Conclusions

Multiple sclerosis should be considered in the differential diagnosis of patients who present with isolated cranial nerve palsies. Clinicians should have a high index of suspicion when evaluating such patients especially in low prevalence regions close to the equator. Early recognition and treatment of such a “Clinically Isolated Syndrome” may prevent early relapse.

## Consent

Written informed consent was obtained from the patient for publication of this case report and relevant images. A copy of the written consent is available for review by the Editor-in-Chief of International Archives of Medicine.

## Competing interests

The authors declare that they have no competing interests.

## Authors’ contributions

ECR carried out the literature search and drafted the manuscript; RG did the critical revision for important intellectual content in the manuscript and given the final approval of the version to be published; MC and PP helped substantially in the literature search and drafting the manuscript. All authors have read and and approved the final manuscript.
